# Whole-Genome Sequencing of *Micrococcus luteus* Strain Modasa, of Indian Origin

**DOI:** 10.1128/genomeA.00076-13

**Published:** 2013-03-07

**Authors:** A. Ghosh, S. A. Chaudhary, S. R. Apurva, T. Tiwari, S. Gupta, A. K. Singh, K. H. Katudia, M. P. Patel, S. K. Chikara

**Affiliations:** Department of Genomics, Xcelris Labs Ltd, Opp. Satyagrah Chhavani, Bodakdev, Ahmedabad, India

## Abstract

The hydrocarbon-degrading bacterium *Micrococcus luteus* strain Modasa was isolated from contaminated soil from Modasa, North Gujarat, India. Whole-genome sequencing and analysis provide an insight into the potentially important genes responsible for bioremediation.

## GENOME ANNOUNCEMENT

The microbial population is a very diverse group among the living world. Potentially important features of the microbes relate to their versatility in utilizing and degrading large numbers of natural and manmade compounds. This property is highly valuable in bioremediation for the complete degradation and removal of pollutants. A pure culture of *Micrococcus* was isolated from soil (extracted from 2 feet below the soil surface) from Modasa, North Gujarat, India, and was confirmed by Gram staining. Micrococci are Gram-positive cocci that are 0.5 to 3.5 µm in diameter and usually arranged in tetrads or irregular clusters. 16S rRNA genes were sequenced on a 3730xl DNA analyzer at the Xcelris genomic center. The sequences have been submitted to NCBI under accession number JX843779. The culture was identified as *Micrococcus luteus* strain Modasa. The high-quality and high-molecular-weight genomic DNA was isolated using an Xcelgen Bacterial gDNA kit.

The whole-genome sequencing of *Micrococcus luteus* strain Modasa was performed on an Ion Torrent PGM sequencer using a 314 chip. The data generation from the genomic library contained 5,99,985 reads and 75,018,013 nucleotide bases, with an average read length of 125 bp using 100 base chemistry. Assembly using MIRA 3.4.0 generated 281 contigs with a maximum contig size of 50,849 bp and a minimum contig size of 2,503 bp (1). The genome was annotated using the RAST server (Rapid Annotation using Subsystem Technology) (2). In total 2,078 potential coding sequences (CDS) were identified, which included 563 hypothetical genes and 90 RNA genes. Out of 90 RNA genes, 82 coded for tRNA, 4 for rRNA, and 4 for 5S RNA. The GC content of the genome is 73.6%. For metabolic reconstruction, metabolic subsystems were assembled to create a metabolic reaction network for the *Micrococcus luteus* strain Modasa.

The subsystem analysis provided the insight of various genes involved in (i) metabolic pathway cofactors, vitamins, prosthetic groups, and pigmentation; (ii) cell wall and capsule; (iii) virulence, disease, and defense; (iv) metabolism of potassium, sulfur, phosphorus, carbohydrates, nitrogen, protein, DNA, RNA, and aromatic compounds; (v) membrane transport; (vi) iron acquisition and metabolism; (vii) cell division, regulation, and signaling; (viii) stress response; (ix) dormancy and sporulation; (x) fatty acids, lipids, and isoprenoids; and (xi) respiration.

The genus *Micrococcus* is well suited to tolerate and use toxic organic molecules as carbon sources, which enables it to survive in extreme environments. These characteristics make these organisms potentially useful for bioremediation. The basic idea behind bioremediation is to find bacteria that are capable of using the pollutant or contaminant as a food source. Previous studies reveal that catechol 2,3-dioxygenase (3) and various phenyl genes (4, 5) are crucial sources of carbon. The presence of these genes indicates involvement in hydrocarbon degradation for *Micrococcus luteus* strain Modasa isolated from contaminated soil, which gives them an important role in bioremediation.

### Nucleotide sequence accession numbers.

The complete sequence of the *Micrococcus luteus* strain Modasa genome can be accessed under the GenBank accession number AMYK00000000. The version described in this paper is the second version, AMYK02000000.

## References

[1] Masrure A Chayan R Prosenjit P Atima A Ashish G Wriddhiman G 2012 Whole-genome shotgun sequencing of sulfur-oxidatizing chemoautotroph *Pseudamonibacter salicylatoxidans* KCT001. J. Bacteriol. 194:4743–4744 22887656 10.1128/JB.00944-12 PMC3415481

[2] Aziz RK Bartels D Best AA DeJongh M Disz T Edwards RA Formsma K Gerdes S Glass EM Kubal M Meyer F Olsen GJ Olson R Osterman AL Overbeek RA McNeil LK Paarmann D Paczian T Parrello B Pusch GD Reich C Stevens R Vassieva O Vonstein V Wilke A Zagnitko O 2008 The RAST server : rapid annotations using subsystem technology. BMC Genomics 9:75 18261238 10.1186/1471-2164-9-75 PMC2265698

[3] Jyothi K Babu KS Clara NK Kumar A 2012 Identification and isolation of hydrocarbon degrading Bacteria by molecular characterization. Helix 2:105–111

[4] Greenblatt CL Baum J Klein BY Nachshon S Koltunov V Cano RJ Micrococcus luteus—survival in Amber. Microb. Ecol. 48:120–127 15164240 10.1007/s00248-003-2016-5

[5] Salton MR Schmitt MD 1967 Effects of diphenylamine on carotenoids and menaquinones in bacterial membranes. Biochim. Biophys. Acta 135:196–207 6037354 10.1016/0005-2736(67)90114-9

